# Targeted Gold Nanohybrids Functionalized with Folate-Hydrophobic-Quaternized Pullulan Delivering Camptothecin for Enhancing Hydrophobic Anticancer Drug Efficacy

**DOI:** 10.3390/polym13162670

**Published:** 2021-08-10

**Authors:** Sakchai Laksee, Chamaiporn Supachettapun, Nongnuj Muangsin, Pattra Lertsarawut, Thitirat Rattanawongwiboon, Phitchan Sricharoen, Nunticha Limchoowong, Threeraphat Chutimasakul, Tanagorn Kwamman, Kasinee Hemvichian

**Affiliations:** 1 Nuclear Technology Research and Development Center, Thailand Institute of Nuclear Technology (Public Organization), Nakhon Nayok 26120, Thailand; pattra@tint.or.th (P.L.); thitirat@tint.or.th (T.R.); phitchan@tint.or.th (P.S.); threeraphat@tint.or.th (T.C.); tanagorn@tint.or.th (T.K.); kasinee@tint.or.th (K.H.); 2 Program in Petrochemistry and Polymer Science, Faculty of Science, Chulalongkorn University, Bangkok 10330, Thailand; chamai190134@gmail.com; 3 Department of Chemistry, Faculty of Science, Chulalongkorn University, Bangkok 10330, Thailand; nongnuj.j@chula.ac.th; 4 Department of Chemistry, Faculty of Science, Srinakharinwirot University, Bangkok 10110, Thailand; nuntichoo@gmail.com

**Keywords:** gold nanohybrids, nanocarriers, pullulan, camptothecin, hydrophobic anticancer drug, intermolecular interactions, drug release, cytotoxicity, apoptosis

## Abstract

This study presented a green, facile and efficient approach for a new combination of targeted gold nanohybrids functionalized with folate-hydrophobic-quaternized pullulan delivering hydrophobic camptothecin (CPT-GNHs@FHQ-PUL) to enhance the efficacy, selectivity, and safety of these systems. New formulations of spherical CPT-GNHs@FHQ-PUL obtained by bio-inspired strategy were fully characterized by TEM, EDS, DLS, zeta-potential, UV-vis, XRD, and ATR-FTIR analyses, showing a homogeneous particles size with an average size of approximately 10.97 ± 2.29 nm. CPT was successfully loaded on multifunctional GNHs@FHQ-PUL via intermolecular interactions. Moreover, pH-responsive CPT release from newly formulated-CPT-GNHs@FHQ-PUL exhibited a faster release rate under acidic conditions. The intelligent CPT-GNHs@FHQ-PUL (IC_50_ = 6.2 μM) displayed a 2.82-time higher cytotoxicity against human lung cancer cells (Chago-k1) than CPT alone (IC_50_ = 2.2 μM), while simultaneously exhibiting less toxicity toward normal human lung cells (Wi-38). These systems also showed specific uptake by folate receptor-mediated endocytosis, exhibited excellent anticancer activity, induced the death of cells by increasing apoptosis pathway (13.97%), and arrested the cell cycle at the G0-G1 phase. The results of this study showed that the delivery of CPT by smart GNHs@FHQ-PUL systems proved to be a promising strategy for increasing its chemotherapeutic effects.

## 1. Introduction

**Figure 1:**
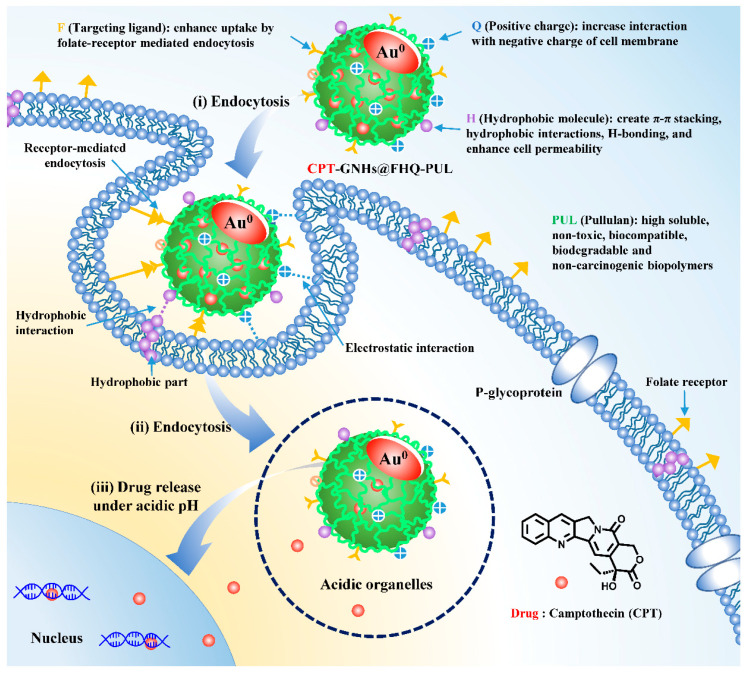
The proposed actions and pH-triggered intracellular release of CPT from CPT-GNHs@FHQ-PUL in cancer cells.

In recent years, lung cancer has become the most common cause of cancer death worldwide [1,2]. General treatments for lung cancer are surgery, radiotherapy, chemotherapy, and the combinations of these treatments. Among various cancer treatments, chemotherapy is the most common type of therapeutic method and consists of the application of anticancer drugs [3]. The major obstacles to effective cancer treatment are related to high toxicity to healthy cells and the acquisition of multidrug resistance [4]. Some of the current challenges that limit the efficacy of many existing chemotherapeutic drugs, especially hydrophobic drugs, include poor solubility and stability in blood, low bioavailability, low anticancer activity, inferior biodistribution in the body, rapid elimination from the body, and potential to cause adverse side effects [3,5]. A hydrophobic anticancer drug, such as camptothecin (CPT), is used to inhibit various cancers, and its chemical structure is shown in Figure 1. CPT is a naturally occurring cytotoxic alkaloid isolated from *Camptotheca acuminata.* CPT displays its activity by inhibiting DNA topoisomerase I, an enzyme that can cleave the supercoil during DNA transcription and replication, forming a covalent enzyme-DNA complex and resulting in single-strand breaks [6]. The applications of CPT in anticancer research (in vitro and in vivo) have been studied to inhibit different cancer cell types, including bladder [7], brain [8], breast [9], cervical [10], colon [11], liver [12], lung [13] and pancreatic cancers [14], by various mechanisms such as growth inhibition of cancer cells, induction of cell cycle arrest and apoptosis, and modulation of inflammation, angiogenesis, and oxidative stress [15]. Nevertheless, CPT is not applied in clinics due to its poor water solubility, low efficacy, non-specificity, severe toxic side effects, and lactone ring instability [16]. Under biological environments, CPT exists either as an active lactone form with high anticancer activity or as an inactive carboxylate form that has higher toxicity to normal cells, restricting the drug oral solubility and bioavailability [17].

Nowadays, significant efforts are currently directed at the development of nanocarriers to solve these problems of unfavorable pharmacokinetic conditions [3]. Several nanocarriers are used in nanomedicine to deliver drugs into the target site via covalent and non-covalent bindings. Two major nanocarriers of inorganic nanocarriers (including silver [18], selenium, iron, silica, and gold nanoparticles (NPs) [19,20,21]) and organic nanocarriers (including polymeric micelles, liposomes, dendrimers, and hydrogels) are broadly used in antimicrobial applications [18] and drug delivery systems which show a variety of complementary properties [22,23,24]. Inorganic nanocarriers are known to provide smaller particle sizes with targeting ability due to unique properties [18] but tend to result in poor biocompatibility, non-biodegradability, and agglomeration of particles [24,25]. On the contrary, organic nanocarriers have high biocompatibility but are able to offer low stability and faster drug release [23]. To address the limitations of each type of nanocarrier, inorganic/organic nanohybrids have been prepared via various techniques [24,26]. Among the broad diversity of nanohybrids (NHs), gold nanohybrids (GNHs) have recently been emerging as promising drug delivery applications for the loading and releasing of drugs [27,28]. GNHs offer better benefits than other metallic NHs because they are inert, non-toxic, and highly biocompatible while concurrently offering unique physicochemical properties [29,30]. In addition, the surface of GNHs can be easily tuned to bind with drugs, and their particle size is easily controlled during preparation by different synthetic methods [24,31].

Recently, green synthesis has attracted considerable attention as a method that uses biopolymers for the synthesis of biopolymer-stabilized GNHs, which can provide effective drug delivery systems, prevent aggregations of GNHs, and reduce the initial burst [32]. In addition, biopolymers, including cassava starch, chitosan, cellulose, hyaluronic acid, and pullulan, have been used in many applications such as agricultural [33], environmental [34], food [35], and biomedical applications [19,36,37]. Among biopolymers, pullulan is a natural biopolymer with non-toxic, non-carcinogenic, biocompatible, biodegradable, and high-soluble properties [38]. However, some aspects have to be considered pertaining to modifications of pullulan, the chemical interactions, and the degree of surface coverage, as these may affect the potential of GNHs [39]. Researchers have studied targeted GNHs by modification of GNHs surface with molecules to increase the selectivity of cancer cells, thereby reducing toxicity, protecting drugs from degradation in human tissues, and sustaining the efficacy of drugs [40,41]. As reported in previous works, the modification of GNHs by cationic molecules, hydrophobic molecules, and folate targeting ligands resulted in electrostatic interactions, hydrophobic interactions, and folate receptor-mediated endocytosis, respectively, which can help to increase the specificity, intracellular uptake, and accumulation of drugs at the target sites [24]. However, to the best of our knowledge, the study of targeted GNHs functionalized with folate-hydrophobic-quaternized pullulan delivering camptothecin (CPT-GNHs@FHQ-PUL) for enhancing drug efficacy has never been reported before.

Thus, the major goal of this study is to develop a green, facile and efficient approach for a new combination of CPT-GNHs@FHQ-PUL to improve the efficacy, selectivity, and safety of these systems. The functional molecules grafted onto PUL-stabilized GNHs can not only create intermolecular interactions with CPT but also offer benefits for increasing uptake, selectivity, and efficacy [42]. For effective drug delivery, PUL was designed to contain three parts, including positive charges, hydrophobic molecules of *p*-aminobenzoic acid, and targeted ligands of folic acid. The first part increased the electrostatic interactions with negatively charged cell membranes, while the second created hydrophobic interactions for enhanced hydrophobic regions of cell membranes [40] and the third increased uptake by receptor-mediated endocytosis [24,43], respectively. After the CPT-GNHs@FHQ-PUL were absorbed, the outer coating of the GNHs with FHQ-PUL provided pH responsiveness and controlled the drug release behaviors. It showed a response to acidic pH to trigger CPT release and further increase the CPT accumulation and activity against human lung cancer cells (Chago-k1), as shown in Figure 1. The cytotoxicity, intracellular uptake, physicochemical property, and mechanism of CPT-GNHs@FHQ-PUL were evaluated by performing apoptosis and cell cycle assays to assess the efficacy of CPT.

## 2. Materials and Methods

### 2.1. Materials

Pullulan (PUL) with an average molecular weight (Mw) of 330 kDa and viscosity of 144.6 mPa-s was purchased from Tokyo Chemical Industry Co., Ltd., Tokyo, Japan. Folate-hydrophobic-quaternized pullulan (FHQ-PUL or FA-PABA-Q188-PUL) was successfully synthesized based on a methodology reported in our previous work [24]. The anticancer drug camptothecin (CPT) was obtained from Sigma-Aldrich, St. Louis, MO, USA.

### 2.2. Preparation of CPT-GNHs@FHQ-PUL

In the preparation process, GNHs were generally synthesized by the green reduction of gold ions (HAuCl_4_ or Au^3+^) to zero-charged GNHs (Au^0^) upon the addition of alkali-treated folate-hydrophobic-quaternized pullulan (FHQ-PUL). After that, CPT-GNHs@FHQ-PUL was prepared by subsequent loading of an anticancer drug (CPT) into GNHs@FHQ-PUL, as described in the following paragraphs. The stock solution of FHQ-PUL was prepared by dissolving 1.2 g of FHQ-PUL powder in 20 mL of milli-Q water. In a typical reaction procedure, 2500 μL of 6.0% *w*/*v* FHQ-PUL solution were mixed with 140 μL of 5.0% *w*/*v* sodium hydroxide solution at 80 °C for 1 h. After the pH was adjusted to 7.0, 1000 μL of 5.0 mM HAuCl_4_ was added to the solution, and the mixture was continuously stirred at 80 °C for 1 h. The color changed from yellow to red, confirming the formation of GNHs@FHQ-PUL. Upon cooling, the solution of GNHs@FHQ-PUL was reconstituted to a final volume of 5.0 mL with milli-Q water. Then, a calculated amount of CPT solution was added to GNHs@FHQ-PUL, resulting in a final CPT concentration of 100 µM. The mixed solution was stirred overnight at room temperature, after which the solution was centrifuged at 10,000 rpm for 20 min. The pellet of GNHs functionalized with FHQ-PUL loading CPT (CPT-GNHs@FHQ-PUL) obtained after centrifugation was separated from the supernatant. The CPT concentration in the supernatant was determined by measuring fluorescence intensity at maximum emission wavelength at 436 nm using a fluorescence spectrophotometer (Agilent Cary Eclipse, Xe pulsed lamp source). Based on the excitation maxima at 380 nm, emission scans were carried out in the range of 400–700 nm with a scan speed of 600 nm/min. The excitation and emission slit widths were kept at 5.0 and 5.0 nm, respectively. To calculate the percent loading efficiency, the following equation (Equation (1)) was used [44]:

% Drug loading efficiency = [(A − B)/A] × 100(1)

where, A is the total amount of CPT added to GNHs@FHQ-PUL and B is the amount of CPT in the supernatant after centrifugation.

### 2.3. Characterization of CPT-GNHs@FHQ-PUL

After preparation, samples were characterized using transmission electron microscopy (TEM) and energy-dispersive X-ray spectroscopy (EDS) for analysis of the morphology, size, shape, and chemical composition of CPT-GNHs@FHQ-PUL. TEM samples were prepared by dropping a solution onto a copper grid. TEM images were recorded on a JEM-2001 instrument operated at 200 kV and equipped with an EDS. The particle size distribution and zeta potential (ζ) distribution of CPT-GNHs@FHQ-PUL were evaluated with a Malvern zeta potential analyzer (Zetasizer nano series version 7.01, Malvern instrument). The GNHs@FHQ-PUL were prepared in milli-Q water at a concentration of 1 mM. The sample size was determined based on the dynamic light scattering (DLS) method and its built-in software. It was collected at an angle of 90° through fiber optics and converted to an electrical signal by an avalanche photodiode. UV-vis spectra of GNHs@FHQ-PUL and CPT-GNHs@FHQ-PUL were characterized on a UV-vis spectrophotometer (HP-8453, Agilent, Santa Clara, CA, USA). UV-vis spectra were measured in the range of 400–900 nm with a scan speed of 600 nm/s. The crystallinity of CPT-GNHs@FHQ-PUL was recorded at 2*θ* in the range of 35–90° using a Bruker AXS D8 ADVANCE X-ray diffractometer with Cu-K_α_ radiation (λ = 1.54056 Å). XRD was set to run at 30 kV and 30 mA in continuous mode with an acquisition step of 0.04° and a scan speed of 1 s/step. The samples were analyzed using a Nicolet-6700 attenuated total reflection-Fourier transform infrared spectrophotometer (ATR-FTIR) at 500–4000 cm^−1^ to characterize CPT-GNHs@FHQ-PUL by identifying the functional groups and the chemical structure. ATR-FTIR spectra were recorded with 32 scans at a resolution of 8 cm^−1^ and collected with Omnic 8.3 data analysis software.

### 2.4. Cell Culture

Three human cancer cell lines, including liver cancer (Hep-G2), gastric cancer (KATO-III), and lung cancer (Chago-k1), were cultured in RPMI-1640 medium with 10% fetal calf serum (FCS) and 1% penicillin-streptomycin solution. Moreover, human lung normal cells (Wi-38) were cultured in a DMEM medium and were supplemented with 10% FCS. After that, cell lines were incubated at 37 °C in 5% carbon dioxide gas (CO_2_).

### 2.5. Cytotoxicity Study

The evaluation of cytotoxicity was based on the reduction of a yellow tetrazolium salt (3-(4,5-dimethylthiazol-2-yl)-2,5-diphenyltetrazolium bromide or MTT) to purple formazan products by NAD(P)H-dependent oxidoreductase enzymes in living cells. Two hundred microliters of cancer cells were seeded into a 96-well plate at a density of 5.0 × 10^3^ cells/well and incubated at 37 °C in 5% CO_2_. After 24 h, the cells were treated with various concentrations of compounds, which were added to triplicate wells for each concentration of each test compound and incubated for 72 h. Next, 10 µL of MTT solution (5 mg/mL) was added to each well, and the mixture was incubated for 4 h. After the medium was removed, the formazan crystals were solubilized in 150 µL of DMSO. The absorbance was measured at 540 nm using a 96-well plate reader, and the obtained value was assumed to represent the number of viable cells. The cell morphology of Chago-k1 cells after treatment with GNHs@FHQ-PUL, CPT, and CPT-GNHs@FHQ-PUL was visualized under a phase-contrast microscope (Nikon DS-L3). The cytotoxic activity was determined as %cell viability. The IC_50_ values, the concentration of an inhibitor that reduces the response by half, were calculated using GraphPad Prism 5 software. The percentage of cell viability was calculated for each compound using Equation (2) [36]:

% Cell viability = (A/B) × 100 (2)

where, A is the absorbance of experimental cells at 540 nm, and B is the absorbance of control cells at 540 nm.

### 2.6. Colony Formation Assay

The colony formation assay was used to evaluate the cell proliferation of cancer cells after long-term treatments with compounds. Chago-k1 cancer cells were placed in a 24-well plate at 1.0 × 10^3^ cells/well for 24 h at 37 °C in a CO_2_ incubator. After that, the cells were treated with various concentrations of compounds. After 10 days, the medium was removed, and cells were washed with phosphate-buffered saline (PBS) solution, fixed with 0.5% glutaraldehyde, and then stained with 0.25% crystal violet solution for 0.5 h. After staining, cells were thoroughly washed with DI water. Colonies were imaged using a phase-contrast microscope (Nikon DS-L3) [24,36].

### 2.7. Intracellular Uptake Study

Chago-k1 cancer cells were plated at a density of 5.0 × 10^3^ cells/well and incubated at 37 °C in a CO_2_ incubator. After 24 h, the cells were treated with 2.5 µM CPT-GNHs@FHQ-PUL at a final GNHs@FHQ-PUL concentration of 40 µM and continuously incubated at 37 °C. After 72 h, the treated cells were harvested, washed, and centrifuged at 2.0 × 10^3^ rpm for 5 min. After that, the treated cells were fixed with 2.5% (*w*/*v*) glutaraldehyde and then with osmium tetroxide prior to being dehydrated in ethanol and then embedded in resin. The sections were sliced off by an ultramicrotome and stained with lead citrate and uranyl acetate. The treated Chago-k1 cells were also observed by TEM to evaluate intracellular uptake of CPT-GNHs@FHQ-PUL compared to the untreated Chago-k1 cells [24,36,44].

### 2.8. Confocal Microscopy

Chago-k1 cancer cells were seeded onto the glass covers at a density of 5.0 × 10^3^ cells/dish in 500 µL of RPMI-1640 medium with 10% FCS. After incubation for 24 h at 37 °C in a CO_2_ incubator, cells were treated with CPT-GNHs@FHQ-PUL at final GNHs@FHQ-PUL and CPT concentrations of 40 and 2.5 µM, respectively. After incubation for 12 h, the medium was removed, and cells were rinsed slowly with PBS solution. After that, the nuclei were stained with 20 µL of DAPI/antifade solution (0.4 mg/mL). After 15 min, the stained cells were then washed with PBS solution to remove excess stain and were imaged with an Olympus FV3000 confocal laser scanning microscope (CLSM) [24,44].

### 2.9. In Vitro Release Behaviors of CPT-GNHs@FHQ-PUL

The release profiles of CPT-GNHs@FHQ-PUL were evaluated at two different pH values by using phosphate saline buffer (PBS at pH 7.4), which represents the pH of the physiological blood and normal tissues in the human body, and acetate buffer at pH 5.0, which represents the average acidity of vesicles in intracellular endosomes and lysosomes (the range of pH = 4.5–6.5). After loading at 100 µM, CPT-GNHs@FHQ-PUL were suspended in 10 mL of PBS at pH 7.4 with constant shaking at 37 °C. At the designated time intervals, a certain volume of the release medium was removed from the tubes for fluorescence analysis. The amount of CPT released in the medium was analyzed using a fluorescence spectrophotometer at 436 nm [24]. A similar release study was conducted in acetate buffer (pH 5.0). The cumulative release of CPT by CPT-GNHs@FHQ-PUL was calculated using Equation (3).

% Cumulative drug release = (A/B) × 100(3)

where, A is the total amount of CPT released at a specific time, and B is the total amount of CPT bound to GNHs@FHQ-PUL.

### 2.10. Cell Cycle Analysis

Chago-k1 cells were seed into 6-well culture plates and incubated at 37 °C in 5% CO_2_ for 24 h. After that, cells were treated with 40 µM GNHs@FHQ-PUL, CPT, and CPT-GNHs@FHQ-PUL at a final CPT concentration of 2.5 and 5.0 µM, respectively. After incubation for 24 h, the treated and untreated cells (1 × 10^6^ cells/mL) were harvested, washed with PBS, and fixed with ice-cold 70% ethanol at −20 °C for 4 h. The fixed cells were washed with PBS and incubated with DNase-free RNase A (0.5 mg/mL) and propidium iodide (PI, 0.02 mg/mL). After 30 min, the data were analyzed using an FC500 flow cytometer (Beckman Coulter, Brea, CA, USA). Acquired data were analyzed using FlowJo V10 software [24,44].

### 2.11. Apoptosis Assay

Chago-k1 cells (2 × 10^6^ cells/well) were seeded into 6-well culture plates and incubated at 37 °C in 5% CO_2_. After 24 h, cells were treated with 40 µM GNHs@FHQ-PUL, CPT, and CPT-GNHs@FHQ-PUL at a final CPT concentration of 2.5 and 5.0 µM, respectively. After incubation for 12 h, cells were harvested, washed with cold PBS, resuspended in Annexin V binding buffer (90 µL, 1X), and incubated with Annexin V-FITC (5 µL) and PI (5 µL) at 0 °C. After 20 min, 400 µL of Annexin V binding buffer (1X) were added and gently mixed. The samples were analyzed using an FC500 flow cytometer. Acquired data were analyzed using FlowJo V10 software [24,44].

## 3. Results and Discussion

### 3.1. Preparation and Characterization of CPT-GNHs@FHQ-PUL

**Figure 2:**
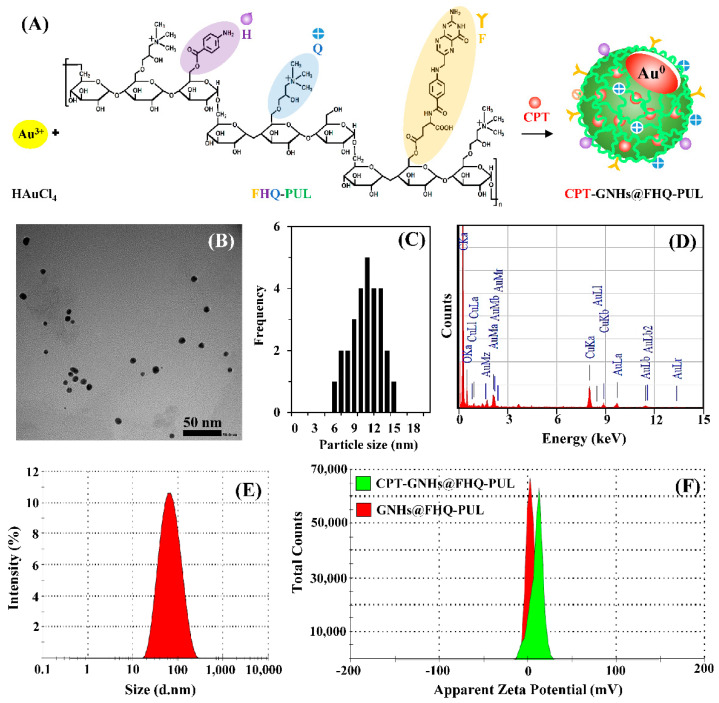
(**A**) Preparation and proposed structure of CPT-GNHs@FHQ-PUL. Characterization of CPT-GNHs@FHQ-PUL: (**B**) TEM image, (**C**) histogram, (**D**) EDS spectrum, (**E**) particle size distribution from DLS analysis, and (**F**) zeta potential distribution.

In this study, CPT-GNHs@FHQ-PUL were successfully prepared by the green reduction of HAuCl_4_ (Au^3+^) into GNHs (Au^0^) with the addition of FHQ-PUL as a trifunctional reducing/capping/stabilizing agent. The proposed mechanism for the anticancer drug delivery systems by the subsequent loading of CPT drug into GNHs@FHQ-PUL via intermolecular interactions is shown in Figure 2A. The system was characterized by TEM, EDS, DLS, zeta potential, UV-vis, XRD, and ATR-FTIR analyses. After the green preparation, the TEM image of CPT-GNHs@FHQ-PUL showed a spherical morphology (Figure 2B). The TEM image revealed dark particles and sphere-shaped particles without aggregation. The particles were well-dispersed and had homogeneous particle sizes with an average size of approximately 10.97 ± 2.29 nm, as shown in the histogram (Figure 2C). The elemental composition of CPT-GNHs@FHQ-PUL was identified by the EDS spectrum (Figure 2D). In addition to characteristic peaks of carbon, oxygen, and copper, the EDS spectrum also showed a strong signal from gold atoms at 7.28% by mass at 2.12 keV. The presence of gold in the EDS spectrum confirmed that the newly formed CPT-GNHs@FHQ-PUL contained gold elements in the GNHs core which were covered by a high concentration of FHQ-PUL shells and CPT (carbon and oxygen atoms). This observation was consistent with the result reported in our previous study [24].

The hydrodynamic diameter of CPT-GNHs@FHQ-PUL dispersed in an aqueous solution was measured by DLS analysis (Figure 2E). It was observed that the average size distribution of the nanohybrids was 51.30 nm which is nearly 4.7 times larger than the size obtained by the TEM image. The hydration and swelling of FHQ-PUL layers at the surface of CPT-GNHs@FHQ-PUL was most likely the main reason for the detection of larger size in DLS analysis. Furthermore, the DLS technique provides the hydrodynamic radius, which is the size of the nanoparticle plus the liquid layer around the nanohybrids, while TEM is only sensitive to the actual size of electron-rich nanohybrids [45]. As shown in Figure 2F, the zeta potential of GNHs@FHQ-PUL was 3.76 mV, whereas CPT-GNHs@FHQ-PUL had a zeta potential of 9.74 mV. After CPT loading and binding, this increase in zeta potential was attributed to attractive forces between CPT and GNHs@FHQ-PUL by intermolecular interactions [46].

**Figure 3:**
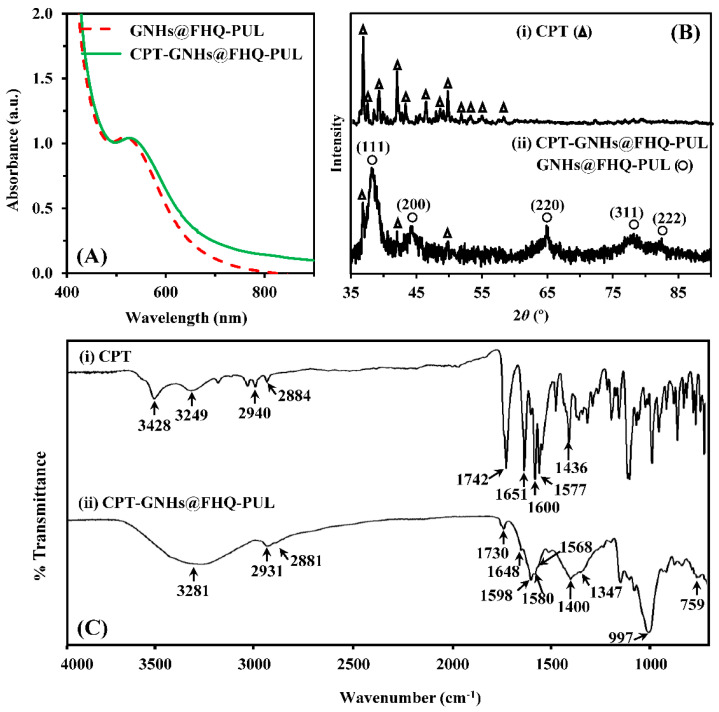
(**A**) UV-vis spectra of GNHs@FHQ-PUL and CPT-GNHs@FHQ-PUL. (**B**) XRD patterns and (**C**) ATR-FTIR spectra of CPT-GNHs@FHQ-PUL compared to CPT.

In addition, the UV-vis spectrum of CPT-GNHs@FHQ-PUL showed a slight redshift from 519 to 528 nm, which confirmed the formation of a protective layer on the GNHs surface upon binding to CPT to form the larger NHs (Figure 3A). The crystalline structure of the incorporated CPT in the GNHs@FHQ-PUL was examined by XRD (Figure 3B).

Intense diffraction peaks of pure CPT (∆) were observed at 2*θ* values of 36.67, 41.84, 49.58°, indicating that CPT has a crystalline nature [47]. Intense and broad diffraction patterns of CPT-GNHs@FHQ-PUL (o) were observed at 2*θ* values of 38.19, 44.41, 64.86, 78.11, and 82.08° corresponding to the (111), (200), (220), (311), and (222) reflection planes of crystalline gold, respectively (JCPDS no.04-0784) [48], whereas new peaks were observed at 2*θ* values of 36.83, 42.08, and 49.81° corresponding to loaded CPT. Less intense peaks with minor shifts of CPT and broad peaks of GNHs@FHQ-PUL indicated that the CPT was well incorporated into GNHs@FHQ-PUL. According to Figure 3C, the ATR-FTIR spectrum of CPT-GNHs@FHQ-PUL demonstrated new characteristic peaks attributed to an ester (C=O), amide (C=O), pyridone ring (C=C), aromatic ring (C=C), and C=N stretching of CPT that shifted from 1742, 1651, 1600, 1577, and 1436 cm^−1^ to 1730, 1648, 1598, 1568, and 1400 cm^−1^, respectively, due to intermolecular interactions between CPT and CPT-GNHs@FHQ-PUL [49]. Furthermore, the drug loading efficiency of the CPT-loaded GNHs@FHQ-PUL was quantified by fluorescence spectroscopy. The loading amount of CPT in the pellets of nanohybrids was calculated using Equation (1) to be 91.4 ± 1.3%, as shown in Supplementary Data (Figure S1). The chemical structure of CPT-GNHs@FHQ-PUL illustrated a high binding affinity by non-covalent bonding, as CPT contains many functional groups that interact with numerous hydroxyl, carboxylate, and phenyl groups of GNHs@FHQ-PUL. FHQ-PUL covered GNHs and produced attractive forces, including hydrogen bonding, π-π stacking, and London dispersion interactions, importantly playing a key role in supporting the spontaneous assembly of GNHs@FHQ-PUL binding with CPT [24]. Consequently, these results confirmed that CPT-GNHs@FHQ-PUL was successfully prepared to form new non-covalently attached drugs that finally lead to the formation of nanohybrid complexes as a lung cancer-targeted delivery system. After the successful preparation, the anticancer activities, intracellular uptake, and mechanism of these systems were studied.

### 3.2. Cytotoxicity Study

GNHs@FHQ-PUL was analyzed for its potential as a hydrophobic drug nanocarrier for improving anticancer activity and the selectivity of the CPT drug against human cancer cells. To the best of our knowledge, the evaluation of the anticancer activity of the drug-loaded nanocarriers (CPT-GNHs@FHQ-PUL) against three human cancer cells and human normal cells by the MTT assay has never been reported before until this study.

**Figure 4:**
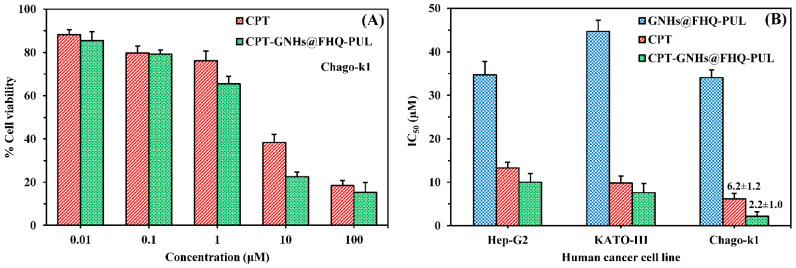
(**A**) % Cell viability in dose-dependent screening of CPT and CPT-GNHs@FHQ-PUL (0.01–100 μM) against Chago-k1 cells. (**B**) Cytotoxicity (IC_50_) of GNHs@FHQ-PUL, CPT, and CPT-GNHs@FHQ-PUL against three human cancer cells. Cytotoxicity was derived after 72 h exposure to NHs by MTT assay. Data are shown as mean ± 1SD, derived from three independent trials.

In terms of cytotoxicity, all samples exhibited dose-dependent within the concentration range of 0.01–100 μM, and the percentage of cell viability of CPT-GNHs@FHQ-PUL and CPT decreased significantly with increasing concentration of these systems, as shown in Figure 4A. The ability of CPT-GNHs@FHQ-PUL to inhibit the cell growth of Chago-k1 cells was obviously better and more effective than that of CPT, agreeing very well with results reported in our previous study [24]. When the incubation time increased from 24 to 72 h, the cytotoxicity of GNHs@FHQ-PUL increased due to higher inhibition of cell growth. In addition, the anticancer activity of GNHs@FHQ-PUL as a nanocarrier at 72 h demonstrated the best inhibition of Chago-k1 growth compared with other incubation times (Figure S2). Among these cancer cells, the prepared CPT-GNHs@FHQ-PUL showed high cytotoxicity toward Chago-k1 cells and was more sensitive than free CPT, as illustrated in Figure 4B. At the same time, the GNHs@FHQ-PUL nanocarriers also showed similarly strong cytotoxic activity against Chago-k1 cancer cells. Herein, the calculated IC_50_ values of free CPT against Hep-G2, KATO-III, and Chago-k1 cancer cells were 13.3 ± 1.3, 9.8 ± 1.6, and 6.2 ± 1.2 μM, respectively. After loading CPT onto the GNHs@FHQ-PUL, the IC_50_ values of these systems toward Hep-G2, KATO-III, and Chago-k1 cancer cells were 10.0 ± 2.0, 7.6 ± 2.1, and 2.2 ± 1.0 μM, respectively. More importantly, the calculated IC_50_ values of CPT-GNHs@FHQ-PUL were 1.30, 1.29, and 2.82 folds lower than the IC_50_ values of free CPT against Hep-G2, KATO-III, and Chago-k1 cancer cells, respectively, due to the enhanced cytotoxicity of CPT mediated by FA-targeted GNHs. Moreover, the biggest difference in IC_50_ between CPT-GNHs@FHQ-PUL and free CPT was observed for human lung cancer cells (Chago-k1). This result implied that GNHs@FHQ-PUL offered the highest efficacy at enhancing intracellular uptake and antiproliferative activity of CPT, most likely due to the loading, binding, and protecting of CPT on GNHs@FHQ-PUL surface modified with targeting ligands, positively charged parts, and hydrophobic parts [50]. This efficacy and selectivity are attributable to the surface-functionalized folate targeting ligand on CPT-GNHs@FHQ-PUL that binds to folate receptors overexpressed on Chago-k1 cancer cells [6,50,51]. Moreover, the positively charged CPT-GNHs@FHQ-PUL increases the electrostatic interactions with the negatively charged cell membranes of Chago-k1 cells. At the same time, the hydrophobic part of CPT-GNHs@FHQ-PUL also creates hydrophobic interactions with hydrophobic parts of Chago-k1 cell membranes, resulting in greater endocytosis and stronger cytotoxic effects [44], as shown in Figure 1. Moreover, the modifications on GNHs@FHQ-PUL not only create the intermolecular interactions with CPT but also enhance intracellular uptake, selectivity, and anticancer efficacy. In the toxicity test against human lung normal cells (Wi-38), CPT-GNHs@FHQ-PUL and GNHs@FHQ-PUL exhibited relatively less toxicity to normal cells with cell viability higher than 80%, while the free CPT was more toxic (Figure S3). Overall, the results suggest that the newly prepared CPT-GNHs@FHQ-PUL systems showed more potent activity in cancer cells while exhibiting less toxicity towards normal cells. Therefore, these systems are promisingly suitable for cancer treatments due to increased selectivity, efficacy, and safety.

### 3.3. Cell Morphology and Colony Formation Assay

**Figure 5:**
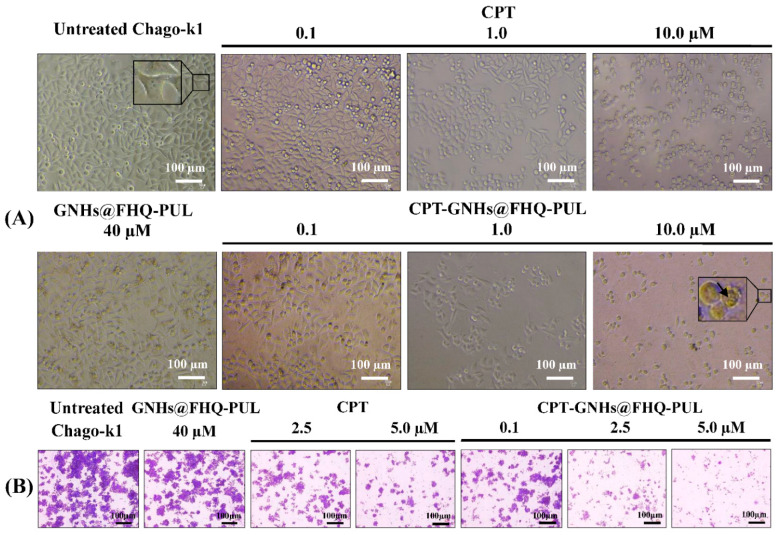
(**A**) Cell morphology and (**B**) colony formation assay of Chago-k1 cells after treatment with GNHs@FHQ-PUL, CPT, and CPT-GNHs@FHQ-PUL at 37 °C visualized under a phase-contrast microscope.

To study the effect of CPT-GNHs@FHQ-PUL on the cell morphology, Chago-k1 cells were treated with CPT-GNHs@FHQ-PUL, CPT, and GNHs@FHQ-PUL. After 72 h, the results were compared with that of untreated Chago-k1 cells, as shown in Figure 5A. Untreated cells showed spindle shape and homogenous cellular contents. The morphology of cells incubated with CPT and GNHs@FHQ-PUL demonstrated circular shape and lower cell density in a dose-dependent manner. As the concentration of CPT-GNHs@FHQ-PUL increased from 0.1 to 10.0 μM, Chago-k1 cells shrunk and changed from spindle to rounder shape, and the density of adherent cells significantly decreased compared to untreated cells and free CPT. These results also suggested that CPT-GNHs@FHQ-PUL strongly induced morphologic damages of Chago-k1 cells. The long-term anticancer activity of CPT-GNHs@FHQ-PUL on colony formation of Chago-k1 cells compared with free CPT and GNHs@FHQ-PUL was also investigated. According to Figure 5B, the CPT-GNHs@FHQ-PUL strongly suppressed colony formation in a dose-dependent manner in Chago-k1 cells, especially at a concentration of 5.0 μM of CPT-GNHs@FHQ-PUL, which exhibited the highest inhibitory activity over free CPT and GNHs@FHQ-PUL, which was also consistent with cytotoxic results by MTT assay.

### 3.4. Intracellular Uptake Study

**Figure 6:**
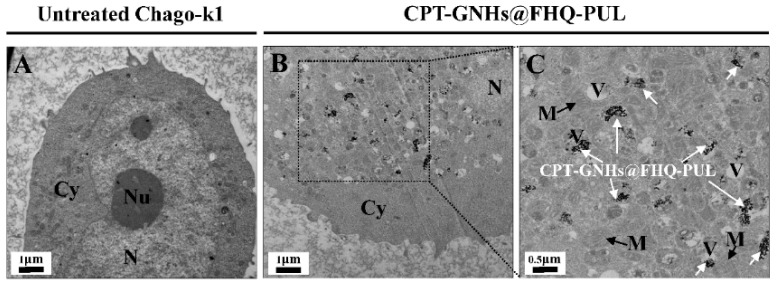
TEM images of Chago-k1 cells after culturing (**A**) without or (**B**,**C**) with CPT-GNHs@FHQ-PUL at 37 °C for 72 h (Nu = nucleolus, N = nucleus, V = vacuole, M = mitochondria and Cy = cytoplasm).

This study was performed to investigate whether CPT-GNHs@FHQ-PUL was taken up in cancer cells by endocytosis. TEM images were used to study intracellular uptake of these systems treatment with or without 2.5 μM CPT-GNHs@FHQ-PUL for 72 h, as shown in Figure 6.

TEM images of untreated Chago-k1 cells exhibited continuous nuclear membranes and cell membranes, whereas cells treated with CPT-GNHs@FHQ-PUL endocytosed and accumulated within the vacuole of cancer cells such as endosomes and lysosomes, resulting in discontinuous nuclear membranes and cell disruption. The intracellular uptake of CPT-GNHs@FHQ-PUL was facilitated by targeting ligands, positively charged parts, and hydrophobic parts on the surface of GNHs. The results indicated that CPT-GNHs@FHQ-PUL as a lung cancer-targeted delivery system could induce intracellular uptake by folate receptor-mediated endocytosis, increasing the cytotoxicity of CPT-GNHs@FHQ-PUL [24].

### 3.5. Confocal Microscopy

**Figure 7:**
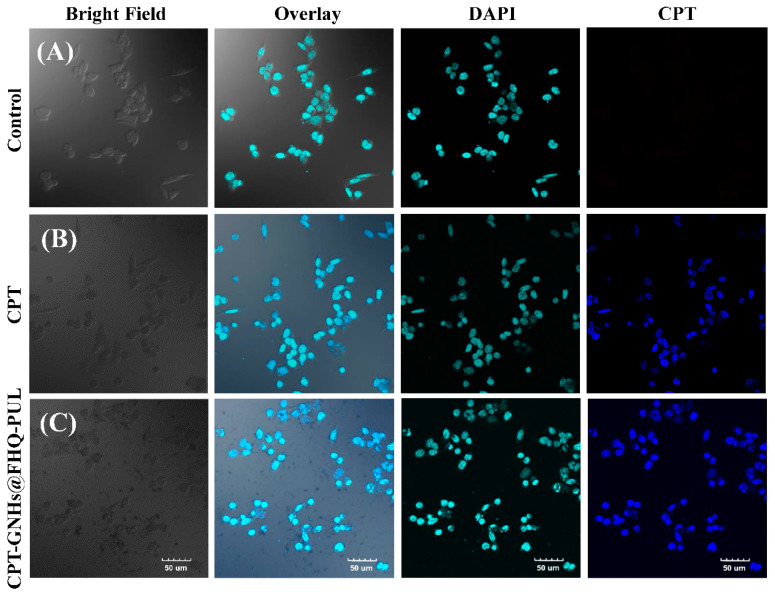
Confocal laser scanning microscopy images of Chago-k1 cells after incubation with (**A**) control, (**B**) CPT, and (**C**) CPT-GNHs@FHQ-PUL at 37 °C for 12 h.

A confocal laser scanning microscope (CLSM) was also employed to gain more insight into the uptake and localization of CPT-loaded GNHs@FHQ-PUL in Chago-k1 cells. The nuclei of the cells are specifically visualized with DAPI staining (greenish-blue color). From Figure 7, the cells treated with CPT-GNHs@FHQ-PUL exhibited strong blue fluorescence from CPT inside the nucleus of cells. This result implied that CPT was internalized and released from CPT-GNHs@FHQ-PUL at high levels through the reductive environment of the cells. The fluorescence signals in cells treated with CPT-GNHs@FHQ-PUL were higher than those in cells treated with free CPT and in the control group. These results suggested that CPT-GNHs@FHQ-PUL was taken up by the surface-functionalized GNHs@FHQ-PUL and localized in the cytoplasm. After its release in acidic environments, CPT is promptly diffused into the cells, entering the nucleus by passive diffusion and inhibiting the growth of cancer cells [24,52]. Thus, the enhanced anticancer activity of CPT-GNHs@FHQ-PUL is most probably due to the improved solubility, uptake, and localization of pH-responsive GNHs@FHQ-PUL as multifunctional nanocarriers.

### 3.6. The Release Behaviors of CPT-GNHs@FHQ-PUL

**Figure 8:**
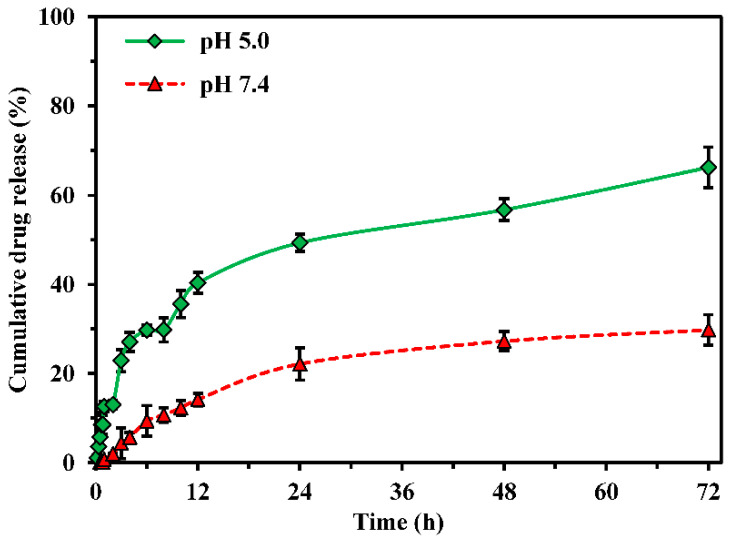
In vitro release profiles of CPT-GNHs@FHQ-PUL in PBS at pH 7.4 and in acetate buffer at pH 5.0.

The in vitro release behaviors of newly prepared CPT-GNHs@FHQ-PUL nanohybrids were explored in PBS at pH 7.4 and in acetate buffer at pH 5.0 to mimic the normal bloodstream and cancer microenvironment [46]. The typical cumulative drug release profiles were illustrated in Figure 8. The cumulative amount of CPT released from CPT-GNHs@FHQ-PUL was calculated by the fluorescence spectra. Under physiological environment, these CPT-GNHs@FHQ-PUL showed a significantly increased CPT release rate at pH 5.0 compared to that at pH 7.4. The cumulative amount of CPT released from the smart nanohybrids at pH 5.0 was 29.75% within 6 h and reached 49.32% at 24 h, while the release rate at pH 7.4 was 9.38% at 6 h and 22.14% at 24 h. At the end of 72 h, 66.21% and 29.79% of CPT were released from these smart nanohybrids in acetate buffer and PBS buffer, respectively. Hence, the release of CPT by CPT-GNHs@FHQ-PUL at acidic pH was better than at neutral pH, so a more acidic microenvironment stimulated the swelling of GNHs@FHQ-PUL and controlled the release of CPT [24]. The pH-responsive nanohybrids provide selective drug release at acidic intracellular vesicles such as lysosomes and endosomes in the cancer cells. After nanohybrids are taken up into the cells, these systems will be responsive to acidic pH for selective release and accumulation of CPT in cancer cells away from P-gp located on cell membranes, resulting in increased anticancer activity in the human lung cancer cells. Results from this study presented good NHs for the slow-release behavior of CPT from the CPT-GNHs@FHQ-PUL, which can prevent limitations of CPT from enhancing solubility and stability of the active lactone form by encapsulation in GNHs@FHQ-PUL. The release of half of CPT at pH 7.4 was highly beneficial to decrease the inactive carboxylate form and the toxicity of CPT to normal human cells [3], corresponding very well with the MTT results.

### 3.7. Cell Cycle Analysis

**Figure 9:**
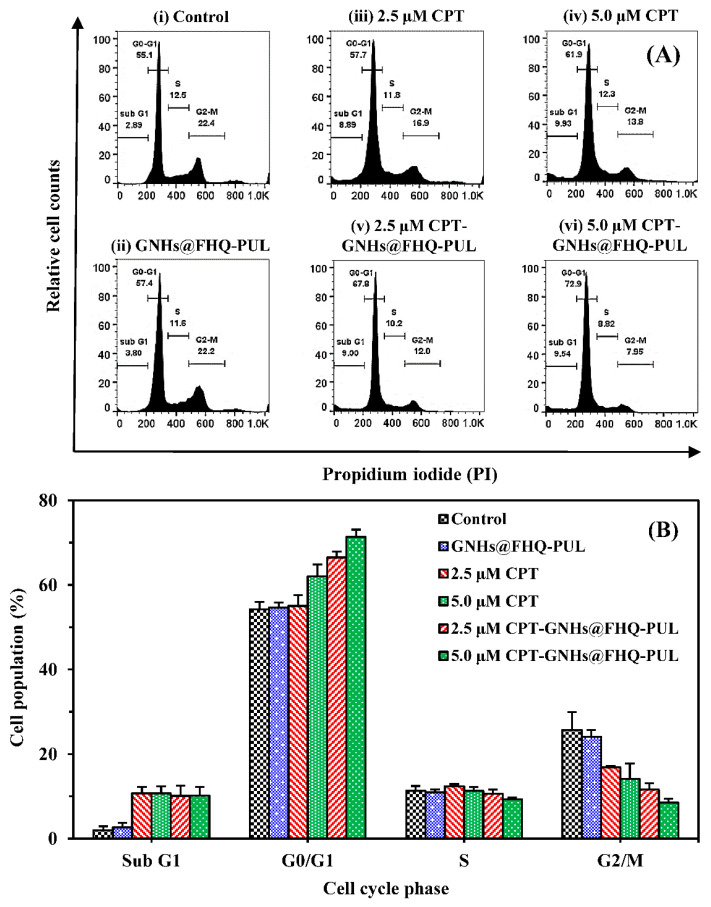
(**A**) Cell cycle analysis and (**B**) cell populations of Chago-k1 cells after treatment with (**i**) control, (**ii**) GNHs@FHQ-PUL, (**iii**,**iv**) CPT, or (**v**,**vi**) CPT-GNHs@FHQ-PUL at 37 °C for 24 h.

The analysis of the cell cycle population of Chago-k1 cells treated with the CPT-GNHs@FHQ-PUL for 24 h and stained using PI, which was detected by flow cytometer, was performed. Figure 9 exhibits changes in the distribution of different cell cycle phases with CPT-GNHs@FHQ-PUL concentration [53]. The cell percentage in gap 0-gap 1 phase (G0-G1) increased after treatment with CPT-GNHs@FHQ-PUL, while cell populations in DNA synthesis phase (S) and gap 2-mitosis phase (G2-M) sharply decreased in the range 2.5–5.0 μM of CPT-GNHs@FHQ-PUL. No significant changes were observed in cell cycle distribution after treatment with GNHs@FHQ-PUL at the same concentration compared to the control group. The numbers of cell population in the G0-G1 phase in the group incubated with 5.0 µM CPT-GNHs@FHQ-PUL significantly enhanced to approximately 17.80%, and the number of cells in S and G2-M phases reduced to 3.68% and 14.45%, respectively, compared to the control group (Figure 9A). Similarly, cells were treated with a decreased concentration of 2.5 µM CPT-GNHs@FHQ-PUL. The newly prepared CPT-GNHs@FHQ-PUL significantly increased the number of cell population in the G0-G1 phase by 9.33 ± 1.14 to 11.43 ± 1.21% compared with free CPT at the same dose (Figure 9B). Hence, the abrogation of the S and G2-M phases arrest caused the accumulation in G0/G1 phase [54]. These data validated the fact that CPT-GNHs@FHQ-PUL induced cell cycle arrest at the G0-G1 phase, and the mode of action for CPT was to suppress DNA synthesis during the cell cycle progression through the inhibition of topoisomerase I [55]. Results from this study confirmed that CPT-GNHs@FHQ-PUL significantly increased the cell cycle disruption at phase G0-G1, resulting in Chago-k1 cell apoptosis.

### 3.8. Apoptosis Assay

**Figure 10:**
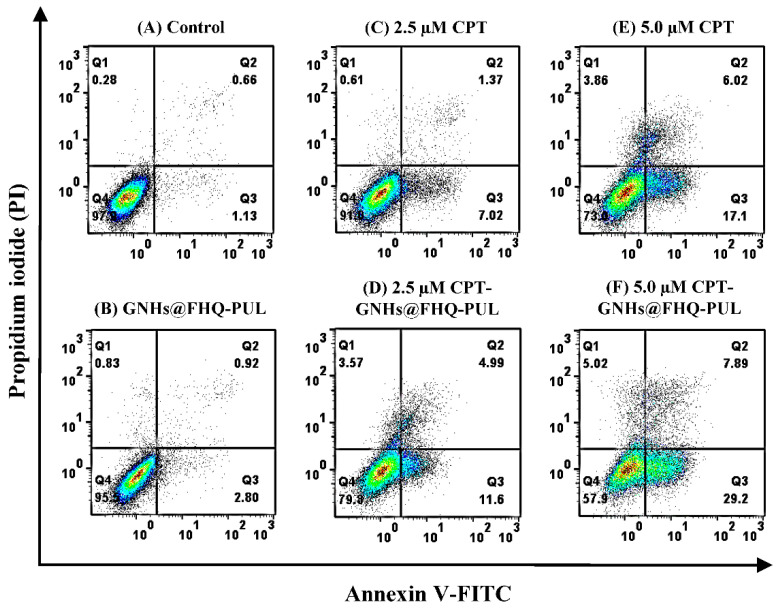
Flow cytometric analysis of Chago-k1 cells after treatment with (**A**) control, (**B**) GNHs@FHQ-PUL, (**C**,**D**) CPT, or (**E**,**F**) CPT-GNHs@FHQ-PUL at 37 °C for 12 h, as detected by Annexin V-FITC/PI staining assay (Q1 represents necrotic cells, Q2 represents late apoptotic cells, Q3 represents early apoptotic cells and Q4 represents live cells).

For anticancer drugs, cancer-related apoptosis is also a key activity to get the best strategy in anticancer therapy [46,56]. Based on the significantly decreased viability of Chago-k1 cancer cells after treatment with CPT-GNHs@FHQ-PUL (Figure 4), the effect of CPT-GNHs@FHQ-PUL on cell apoptosis was evaluated in their cytotoxic mechanism using Annexin V-FITC/PI dual staining assay. The representative areas showing apoptotic (Annexin V^+^) or necrotic cells (Q1, Annexin V^−^/PI^+^) from different groups were detected by flow cytometer. As illustrated in Figure 10, the obtained results showed that 97.9% and 95.5% of untreated Chago-k1 cells in the control group and cells treated with GNHs@FHQ-PUL were viable after 12 h, respectively, whereas free CPT and 5.0 μM CPT-GNHs@FHQ-PUL treatments resulted in 73.0% and 57.9% of viable cells, as shown in Q4 (Annexin V^−^/PI^−^). The findings displayed a significant enhancement in the percentage of both early (Q3, Annexin V^+^/PI^−^) and late (Q2, Annexin V^+^/PI^+^) apoptotic cells in a CPT-GNHs@FHQ-PUL concentration-dependent manner. Importantly, 37.09% of total apoptotic cells after treatment with 5.0 μM CPT-GNHs@FHQ-PUL was observed, which remarkably increased the total percentage of apoptotic cells by 13.97% compared to free CPT (5.0 μM, 23.12%). Similarly, cells were treated with a decreased concentration of 2.5 µM CPT-GNHs@FHQ-PUL, consistent with the results from MTT assay and intracellular uptake. Based on these results, CPT-GNHs@FHQ-PUL importantly increased the number of early and late apoptotic cells to a greater extent than free CPT. Consequently, these results indicated that GNHs@FHQ-PUL served as multifunctional nanocarriers to deliver both hydrophobic and hydrophilic drugs [24], offering the high potential to induce apoptosis of human lung cancer cells and great promise to decrease the side effects of drugs [57]. Therefore, the new combination of CPT-GNHs@FHQ-PUL as targeted nanocarriers improved the physicochemical characteristics while simultaneously promoting the anticancer efficiency of CPT and exerting more profound effects on treating human cancer.

## 4. Conclusions

In conclusion, this study demonstrated that GNHs@FHQ-PUL was proved to be a promising multifunctional nanocarrier that can deliver CPT as a hydrophobic anticancer drug into human lung cancer cells (Chago-k1). Monodispersed CPT-GNHs@FHQ-PUL showed a strong LSPR band at 528 nm with an average size of approximately 10.97 ± 2.29 nm. Smart GNHs@FHQ-PUL was compatible with CPT, forming self-assembly via intermolecular interactions. The anticancer activities of newly prepared CPT-GNHs@FHQ-PUL were evaluated and found to enhance the activity of CPT by approximately 2.82-fold, resulting in reduced IC_50_ values compared to CPT alone. CPT-GNHs@FHQ-PUL were taken up into the cells by folate receptor-mediated endocytosis, hydrophobic interactions, and electrostatic interactions. TEM and CLSM images revealed that CPT-GNHs@FHQ-PUL were dispersed in the cytoplasm, accumulating around the nucleus. The release behaviors of CPT-GNHs@FHQ-PUL nanohybrids were pH-dependent, offering a faster release of CPT from these systems at pH 5.0, compared to pH at 7.4. The results from this study suggested that these systems induced cell apoptosis by increasing both early and late apoptosis cells and arrested the cell cycle at the G0-G1 phase. Therefore, it is inferred that the effective drug delivery system of smart CPT-GNHs@FHQ-PUL offers a promising potential to be used for chemotherapy.

## Data Availability

The data presented in this study are available upon request.

## Supplementary Materials

The following are available online at https://www.mdpi.com/article/10.3390/polym13162670/s1: Figure S1: (A) Fluoresces spectra and (B) % drug loading efficiency of CPT-GNHs@FHQ-PUL compared with CPT-GNHs@PUL; Figure S2: (A) % Cell viability of human lung cancer cells (Chago-k1) after incubation with GNHs@FHQ-PUL (50 and 100 μM) at 37 °C for 24, 48 and 72 h. Data are shown as mean ± 1SD of triplicate experiments; Figure S3: (A) % Cell viability of human lung normal cells (Wi-38) after treatment with 40 μM GNHs@FHQ-PUL, 5 μM CPT and 5 μM CPT-GNHs@FHQ-PUL at 37 °C for 72 h. Data are shown as mean ± 1SD, derived from three independent trials.
