# Supplementary material for: Targeted Gold Nanohybrids Functionalized with Folate-Hydrophobic-Quaternized Pullulan Delivering Camptothecin for Enhancing Hydrophobic Anticancer Drug Efficacy
Source: Polymers (Basel). 2021 Aug 10;13(16):2670. doi: 10.3390/polym13162670 (PMC8400492; doi:10.3390/polym13162670)
Supplement: Supplementary file 1 [file polymers-13-02670-s001.zip › polymers-1321896-supplementary.pdf]

polymers

Article

# Targeted Gold Nanohybrids Functionalized with Folate-Hydrophobic-Quaternized Pullulan Delivering Camptothecin for Enhancing Hydrophobic Anticancer Drug Efficacy

**Sakchai Laksee [1,*], Chamaiporn Supachettapun [2], Nongnuj Muangsin [3], Pattra Lertsarawut [1], Thitirat Rattanawongwiboon [1], Phitchan Sricharoen [1], Nunticha Limchoowong [4], Threeraphat Chutimasakul [1], Tanagorn Kwamman [1] and Kasinee Hemvichian [1]**

[1] Nuclear Technology Research and Development Center, Thailand Institute of Nuclear Technology (Public Organization), Nakhon Nayok 26120, Thailand; pattra@tint.or.th (P.L.); thitirat@tint.or.th (T.R.); phitchan@tint.or.th (P.S.); threeraphat@tint.or.th (T.C.); tanagorn@tint.or.th (T.K.); kasinee@tint.or.th (K.H.)
[2] Program in Petrochemistry and Polymer Science, Faculty of Science, Chulalongkorn University, Bangkok 10330, Thailand; chamai190134@gmail.com
[3] Department of Chemistry, Faculty of Science, Chulalongkorn University, Bangkok 10330, Thailand; nongnuj.j@chula.ac.th
[4] Department of Chemistry, Faculty of Science, Srinakharinwirot University, Bangkok 10110, Thailand; nuntichoo@gmail.com
* Correspondence: sakchai@tint.or.th

**Citation:** Laksee, S.; Supachettapun, C.; Muangsin, N.; Lertsarawut, P.; Rattanawongwiboon, T.; Sricharoen, P.; Limchoowong, N.; Chutimasakul, T.; Kwamman, T.; Hemvichian, K. Targeted Gold Nanohybrids Functionalized with Folate-Hydrophobic-Quaternized Pullulan Delivering Camptothecin for Enhancing Hydrophobic Anticancer Drug Efficacy. *Polymers* **2021**, *13*, 2670. https://doi.org/10.3390/polym13162670

Academic Editor: Iole Venditti

Received: 15 July 2021
Accepted: 7 August 2021
Published: 10 August 2021

**Publisher's Note:** MDPI stays neutral with regard to jurisdictional claims in published maps and institutional affiliations.

**Copyright:** © 2021 by the authors. Licensee MDPI, Basel, Switzerland. This article is an open access article distributed under the terms and conditions of the Creative Commons Attribution (CC BY) license (http://creativecommons.org/licenses/by/4.0/).

## Supplementary Information

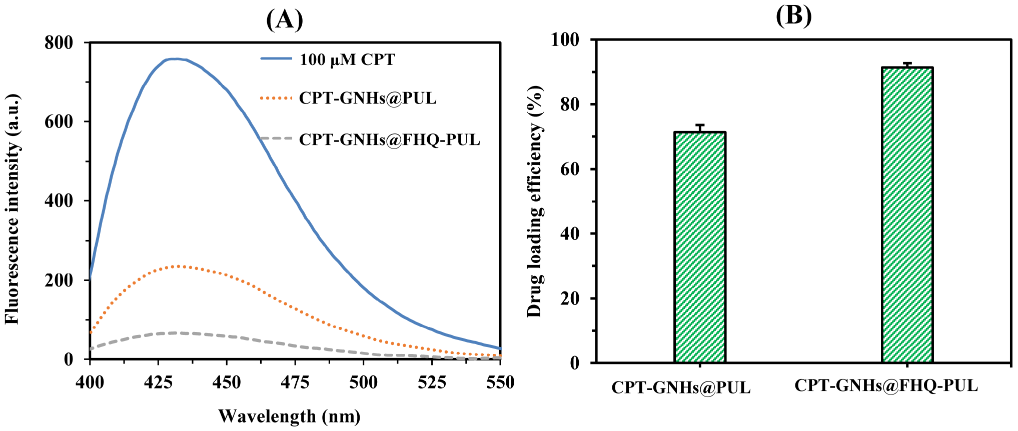


**Figure S1.** (**A**) Fluoresces spectra and (**B**) % drug loading efficiency of CPT-GNHs@FHQ-PUL compared with CPT-GNHs@PUL.

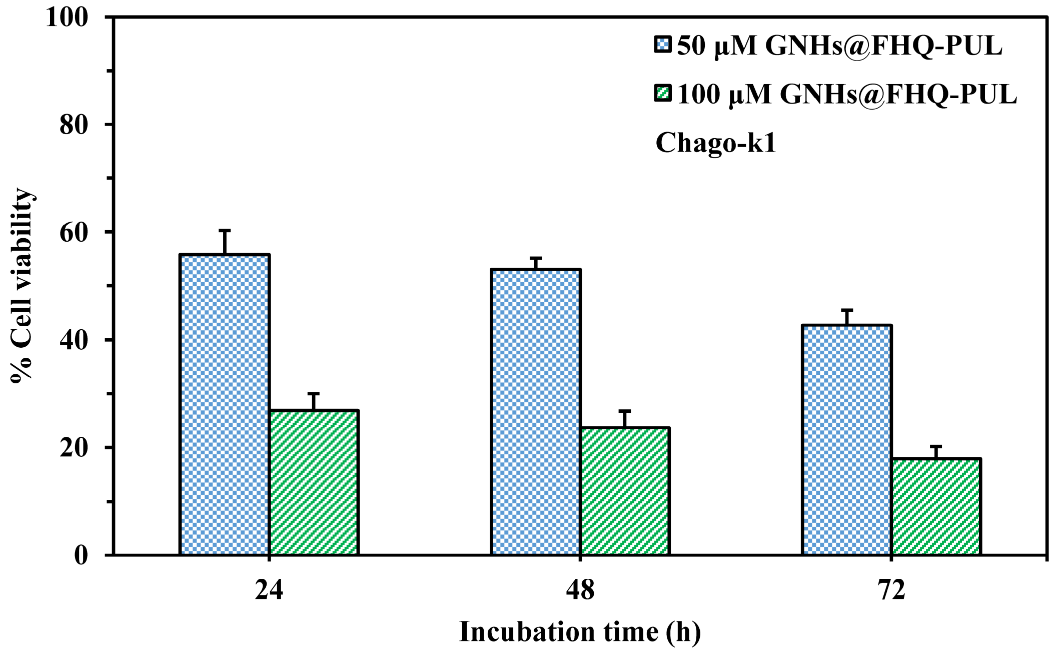


**Figure S2.** (A) % Cell viability of human lung cancer cells (Chago-k1) after incubation with GNHs@FHQ-PUL (50 and 100 µM) at 37 ºC for 24, 48 and 72 h. Data are shown as mean ± 1SD of triplicate experiments.

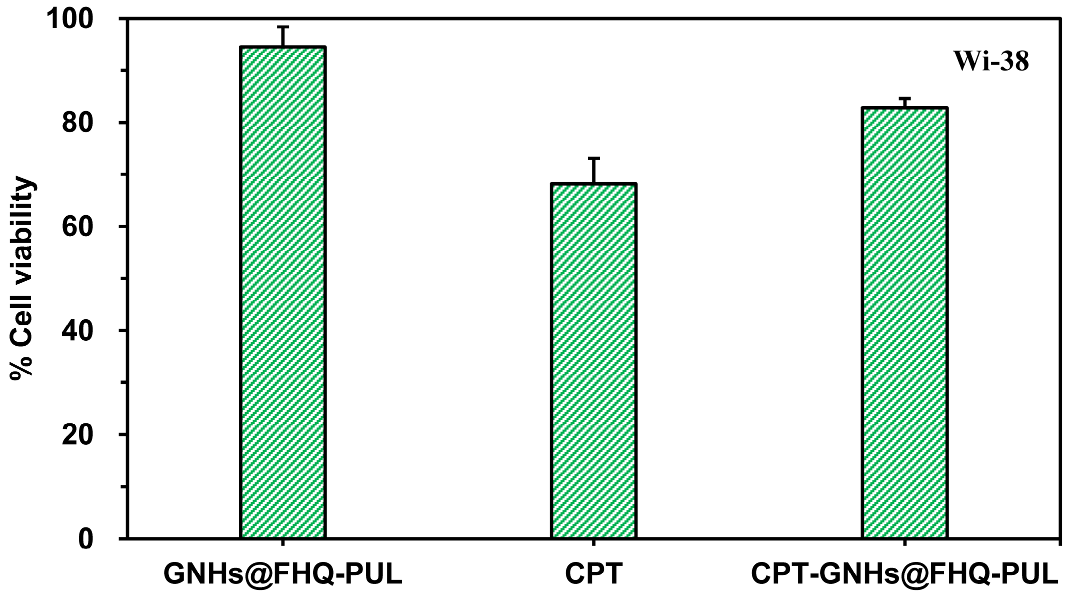


**Figure S3.** (A) % Cell viability of human lung normal cells (Wi-38) after treatment with 40 µM GNHs@FHQ-PUL, 5 µM CPT and 5 µM CPT-GNHs@FHQ-PUL at 37 ºC for 72 h. Data are shown as mean ± 1SD, derived from three independent trials.
